# Revised nomenclature and functional overview of the ULP gene family of plant deSUMOylating proteases

**DOI:** 10.1093/jxb/ery301

**Published:** 2018-08-14

**Authors:** Pedro Humberto Castro, Andreas Bachmair, Eduardo R Bejarano, George Coupland, L Maria Lois, Ari Sadanandom, Harrold A van den Burg, Richard D Vierstra, Herlander Azevedo

**Affiliations:** 1 CIBIO, InBIO – Research Network in Biodiversity and Evolutionary Biology, Universidade do Porto, Vairão, Portugal; 2 Dept of Biochemistry and Cell Biology, Max F. Perutz Laboratories, University of Vienna, Vienna, Austria; 3 Area de Genética, Instituto de Hortofruticultura Subtropical y Mediterránea ‘La Mayora’, Universidad de Málaga-Consejo Superior de Investigaciones Científicas (IHSM-UMA-CSIC), Málaga, Spain; 4 Max Planck Institute for Plant Breeding Research, Köln, Germany; 5 Center for Research in Agricultural Genomics-CRAG, Edifici CRAG-Campus UAB, Bellaterra (Cerdanyola del Vallés), Barcelona, Spain; 6 Durham Centre for Crop Improvement Technology, Department of BioSciences, Durham University, Durham, United Kingdom; 7 Molecular Plant Pathology, Swammerdam Institute for Life Sciences, University of Amsterdam, Amsterdam, The Netherlands; 8 Department of Biology, Washington University in St Louis, St Louis, Missouri, USA; 9 Departamento de Biologia, Faculdade de Ciências, Universidade do Porto, Porto, Portugal

**Keywords:** Arabidopsis, deSUMOylating protease, nomenclature, SUMO protease, sumoylation, ULP

**Functional insight on the post-translational modifier SUMO and its biochemical pathway in plants has steadily increased over the past decade. In contrast to the low number of core components that catalytically control SUMO attachment to targets, the enzymes that control deconjugation and SUMO maturation seem to have diversified in terms of both gene number and biological function. However, studies on these deSUMOylating proteases have been accompanied by diversity in nomenclature and unclear evolutionary categorization. We provide a state-of-the-art assessment of the evolutionary subclades within the ULP gene family of plant deSUMOylating proteases, and propose a nomenclature for this protease subgroup for consistent annotation of ULP-encoding genes in plant genomes.**

The Small Ubiquitin-like Modifier (SUMO) polypeptide is a member of the Ub-fold family, which is collectively defined by a signature β-grasp fold. Like ubiquitin (Ub), SUMO acts in the post-translational modification of proteins, and is important for plant development and adaptive responses to the environment (Castro *et al.*, 2012; Yates *et al.*, 2016). The SUMO conjugation and deconjugation cycles have to be tightly regulated, and numerous SUMO proteases are fundamental for this equilibrium. Several types of deSUMOylating proteases (DSPs) were uncovered in non-plant models, namely ULP/SENPs, DESIs and USPLs, which belong to separate families of cysteine proteases (C48, C97 and C98, respectively) (Hickey *et al.*, 2012; Nayak and Muller, 2014). Presently, the only functionally characterized plant DSPs belong to the Ub-Like Protease (ULP) gene family.

## Evolution and nomenclature in plant ULPs

ULPs are cysteine proteases belonging to the C48 family (MEROPS release 12.0; Rawlings *et al.*, 2018). Despite sharing similarities with the catalytic domains of some classes of deubiquitylating proteases, such as Ubiquitin Specific Proteases (UBPs) and Ubiquitin C-terminal Hydrolases (UCHs), they belong to different clans (clan CE for ULPs, and clan CA for UBPs and UCHs). CE and CA proteases share a papain-like fold and, most likely, a common origin (van der Hoorn, 2008; Rawlings *et al.*, 2018). Historically, ULPs have been divided into two large groups (ULP1s and ULP2s), following the identification of two functionally separate paralogs – ScULP1 and ScULP2/Smt4 in yeast (Li and Hochstrasser, 1999, 2000). Later, human ULPs were also differentiated into ULP1s (SENP1, -2, -3 and -5), and ULP2s (SENP6 and -7) (Mukhopadhyay and Dasso, 2007). Plant deSUMOylating proteases belonging to the ULP gene family have mostly been studied in the model plant Arabidopsis. Despite the significant functional advances, difficulties have arisen in establishing definitive gene abundance, phylogeny and nomenclature of this gene family.

### Gene abundance

The Arabidopsis genome is assumed to contain eight ULPs (Box 1) (Novatchkova *et al.*, 2012; Castro *et al.*, 2016; Benlloch and Lois, 2018; Garrido et al., 2018). Often, however, only seven have been described because of the failure to incorporate At3g48480 (Novatchkova *et al.*, 2004; Colby *et al.*, 2006; Hoen *et al.*, 2006), as this is a highly truncated form albeit one that retains the protease domain. Also, initial phylogenetic studies incorporated At5g60190 (Novatchkova *et al.*, 2004; Hoen *et al.*, 2006), which was subsequently identified as a deNEDDylating rather than a deSUMOylating protease, and named Deneddylase 1 (DEN1; Box 1) (Colby *et al.*, 2006; Mergner *et al.*, 2015). Initial reports similarly established a massive gene expansion in this gene family (Kurepa *et al.*, 2003; Hoen *et al.*, 2006; Lois, 2010). This has been traced to the presence of at least 97 MULE transposons that contain intact peptidase C48 domains, and are likely to have expanded via ancient transduplication events (Hoen *et al.*, 2006). Though these amplified genomic loci may encode polypeptides that possess SUMO protease activity, they are phylogenetically more distant than the deNEDDylating protease DEN1 when compared to ULPs, and display low or undetectable expression, which suggests they are unlikely to act towards SUMO (Hoen *et al.*, 2006). Hoen and co-workers (2006) have named these Kaonashi (KI) elements, and here we propose a definitive nomenclature as Kaonashi ULP Like Proteases (KIUs) (Box 1).

**Box 1. Plant ULP evolution and nomenclature**

- A schematic tree, depicting currently accepted phylogenetic relationships between organisms, summarizes the evolutionary path of the plant ULP gene family of deSUMOylating proteases. Plant ULPs have a polyphyletic origin than can be traced to green algae and ultimately to examples in other eukaryotes, including ScULP1 and ScULP2. ULP1s form a homogenous class (Class I, ELS-type), while ULP2s branch out into Class II (OTS-type) and Class III (SPF-type) proteases during early plant evolution. Class IV (FUG-type) consistently appears in flowering plant genomes and seems absent from early plant taxa, but its origin remains elusive (Castro *et al.*, 2018).
- Existing nomenclature for all Arabidopsis ULPs. We propose a nomenclature that reflects biological function and assumed phylogenetic relationships. It incorporates new gene names for two Arabidopsis ULPs (highlighted in blue). In future annotation of plant genomes, plant ULPs may be spelled with a prefix of the species, followed by increasing numbering. For example, tomato Class II ULPs may be named SlOTS1, SlOTS2, and so on. References in main text; see also Miura *et al.*, 2007.

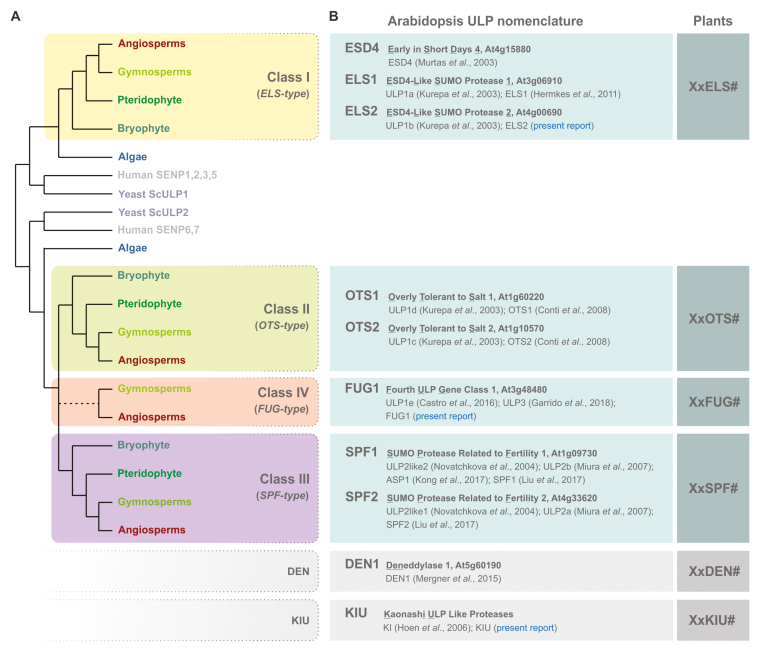

### Gene phylogeny

The eight canonical Arabidopsis ULPs have consistently been categorized in light of their strong amino acid sequence conservation to yeast ULP1 or ULP2 (Kurepa *et al.*, 2003; Novatchkova *et al.*, 2004; Mukhopadhyay and Dasso, 2007; Lois, 2010), though they can be resolved into additional phylogenetic subgroups (Colby *et al.*, 2006; Novatchkova *et al.*, 2012) (Box 1). Insight based on more extensive comparative genomics data suggests that At4g15880/At3g06910/At4g00690 form a homogenous class of ULP1s (homologous to yeast ScULP1). In contrast, Arabidopsis homologs of ScULP2 can be divided into three classes, containing At4g33620/At1g09730, At1g10570/At1g60220 and At3g48480 (Novatchkova *et al.*, 2012; Castro *et al.*, 2018). Existence of four classes is also supported by protein topological data, namely protein size and the location of the ULP domain (Benlloch and Lois, 2018; Castro *et al.*, 2018). Here, we propose a definitive classification for the four plant ULP classes (Classes I–IV) based on the Arabidopsis ULPs (Box 1).

### Gene nomenclature

The community has been struggling to define a coherent naming of Arabidopsis ULPs. Initially they were named after assumed phylogenetic relatedness to ULP1 or ULP2 proteins. Erroneously, this led to the naming of At1g10570, At1g60220 and At3g48480 as ULP1c, ULP1d and ULP1e, respectively (Kurepa *et al.*, 2003; Lois, 2010; Castro *et al.*, 2016), even though they are phylogenetically related to ULP2s. Functional studies in Arabidopsis generated an increasing number of names that disregarded molecular function in favor of biological function, resulting in several parallel nomenclatures. Most ULP genes have between two and as many as four names for a single member. It is important to clarify this matter to create a consensual nomenclature based on biological function, while at the same time respecting known phylogenetic data. The proposed nomenclature is detailed in Box 1.

## ULP function

It is well established in non-plant models that ULPs are regulated at various levels, including enzymatic activity, SUMO isoform discrimination, subcellular localization and expression patterns (Hickey *et al.*, 2012; Nayak and Muller, 2014; Kunz *et al.*, 2018). A series of clues point towards similarly complex functionalities for plant ULPs. Characterization of loss-of-function Arabidopsis ULP mutants has implicated the different ULP classes in non-redundant functions during plant development. The *esd4* mutant has a pleiotropic phenotype accompanied by early flowering, partially due to SA accumulation (Murtas *et al.*, 2003; Villajuana-Bonequi *et al.*, 2014), while loss-of-function of its closest paralog ELS1 does not display such a drastic phenotype (Hermkes *et al.*, 2011). OTS mutants assume a mild developmental phenotype (smaller and early-flowering plants), and are also implicated in abiotic and biotic stress resistance (Conti *et al.*, 2008; Bailey *et al.*, 2016; Castro *et al.*, 2016). In contrast, SPF-class mutants are late flowering, and display an altered growth pattern and embryo development defects (Kong *et al.*, 2017; Liu *et al.*, 2017; Castro *et al.*, 2018). The fourth class of ULPs, represented in Arabidopsis by *FUG1*, is yet to be functionally addressed. Future studies may bring to light additional deSUMOylating protease gene families other than ULPs, adding complexity to the SUMO pathway.

As previously established for non-plant ULPs, different subcellular targeting is an important aspect of ULP molecular function (Hickey *et al.*, 2012; Nayak and Muller, 2014; Kunz *et al.*, 2018). In Arabidopsis, ESD4 interacts with the nuclear pore component NUA, which concentrates its location at the inner nuclear side of the nuclear pore (Xu *et al.*, 2007). In contrast, ELS1 resides in the cytoplasm, which supports low functional redundancy between Class I proteases in Arabidopsis (Hermkes *et al.*, 2011). OTS1, OTS2, SPF1 and SPF2 are nuclear proteins (Conti *et al.*, 2008; Liu *et al.*, 2017; Castro *et al.*, 2018). With the possible exception of the functionally uncharacterized genes *ELS2* and *FUG1*, Arabidopsis ULPs are widely expressed. In classes I and II, there is one ULP that is more expressed than the remaining class members (*ESD4* and *OTS1*, respectively). *OTS1* and *OTS2* seem to display similar expression patterns but differences in expression amplitude, while *SPF1* and *SPF2* show differential expression patterns, collectively explaining the existence of unequal functional redundancy in these gene pairs (Castro *et al.*, 2016; Liu *et al.*, 2017; Castro *et al.*, 2018).

## Further research on plant deSUMOylating proteases

Our understanding of the functions of deSUMOylation, reviewed more extensively by Benlloch and Lois (2018), is at present very limited. Foremost among future research efforts is determining whether deSUMOylating proteases in general, and ULPs in particular, display a preferential capacity to act as endopeptidases (involved in maturation of preSUMO peptides) or as isopeptidases (removal of SUMOs from SUMO conjugates). Also of significance is the establishment of affinity towards the different SUMO isoforms present in plant genomes, and whether they display capacity to process polySUMO chains. Crystal structure and docking studies of catalytic domains are also needed to complement our analysis of proteolytic activity. The over-representation of ULP gene members in plant genomes in comparison with SUMO conjugation components (Augustine *et al.*, 2016; Castro *et al.*, 2018; Garrido et al., 2018), suggests that ULPs are likely to function, to some extent, as sources of specificity within the SUMO pathway. Proteomics strategies to identify large numbers of SUMO conjugates are progressively being introduced in Arabidopsis SUMO research (Budhiraja *et al.*, 2009; Miller *et al.*, 2010; Lopez-Torrejon *et al.*, 2013; Miller *et al.*, 2013; Rytz *et al.*, 2018). Application of these strategies in ULP mutant backgrounds should help us define the target specificity of these proteases.

As we move away from Arabidopsis to non-model plants, it is important to have a clear vision of ULP function and target specificity, but also of gene abundance and the evolutionary pathway of this gene family. Sound and precise nomenclature should provide a beneficial contribution.
